# Complete Genome Sequence of *Streptococcus pyogenes* M/*emm44* Strain STAB901, Isolated in a Clonal Outbreak in French Brittany

**DOI:** 10.1128/genomeA.01174-14

**Published:** 2014-11-20

**Authors:** Nicolas Soriano, Pascal Vincent, Caroline Piau, Séverine Moullec, Philippe Gautier, Vincent Lagente, Ahmad Faili, Samer Kayal

**Affiliations:** a Département de Microbiologie et Immunologie, Faculté de Médecine et de Pharmacie, Université de Rennes 1, Rennes, France; b Service de Microbiologie et Hygiène hospitalière, CHU Pontchaillou, Rennes, France; c IGDR-UMR 6290-CNRS, Université de Rennes 1, Rennes, France; d UMR991-INSERM, Université de Rennes 1, Rennes, France

## Abstract

We report the complete genome sequence of an invasive isolate of *Streptococcus pyogenes* M/*emm44*, belonging to a clonal outbreak that occurred in French Brittany. The genome is composed of 1,795,608 bp, with a GC content of 38.5%, has 1,358 identified coding sequences (CDSs), and harbors a novel Tn916-like transposon (Tn6253).

## GENOME ANNOUNCEMENT

*Streptococcus pyogenes* strains, or group A streptococci (GAS), are human pathogens responsible for a wide variety of invasive and noninvasive infections (1–3). Strains of GAS are epidemiologically characterized by the sequence of the variable segment of the *emm* gene that encodes the membrane-anchored M protein (2, 3). The occurrence of infections due to M/*emm44* type remains rare (4). We recently reported a clonal spread of a tetracycline-resistant GAS M/*emm44* strain in marginal populations (drug users and homeless persons) (5). Although this clonal profile was mainly associated with poor living conditions and noninvasive infections, it has also been isolated from patients without known risk factors or experiencing severe invasive infection that could be complicated by hemodynamic shock or death. Aiming to decipher the spreading potential and the emerging virulence of this strain we sequenced the genome of the strain STAB901, an invasive GAS M/*emm44* with the same pulsed-field gel electrophoresis (PFGE) profile of our previously described epidemic clone (5). This strain has been isolated from the blood of a patient with endometritis due to an intrauterine contraceptive device and complicated by septic shock.

The STAB901 strain was grown in Todd-Hewitt broth supplemented with 0.2% yeast extract (THY), and DNA was extracted and purified using the phenol-chlorophorm technique. Genomic DNA sequencing was performed using Roche/454 pyrosequencing technology (Genome Sequencer GSFLX system with titanium chemistry) at the Functional Genomics and Environmental Platform (Biogenouest, Rennes, France). By using MIRA3 software (http://www.chevreux.org/projects_mira.html), the whole reads (285,647) were initially assembled in 924 contigs, of which only 16 had a length greater than 10 kb. Based on the available sequences of GAS, these contigs were oriented and ordered and finally assembled using CLC Workbench version 6 software (http://www.clcbio.com). The resulting chromosomal scaffold consisted of 14 separated contigs, and the sequence gaps were filled by PCR and sequencing. The final single circular genome of 1,795,608 bp, with an average G+C content of 38.5%, was fully annotated using the RAST server (6) and NCBI-PGAP (http://www.ncbi.nlm.nih.gov/genome/annotation_prok/). We identified 1,812 genes (67 tRNA, and 18 rRNA) and 1,358 expected coding sequences (CDSs). Using the PHAge Search Tool (PHAST) (7) we identified one putative phage and one intact integrated prophage that harbors the superantigen *speH*. The other chromosomic superantigen genes identified were *speF, speG*, and *speJ*. The multilocus sequence type (MLST) belongs to the ST.178 profile (http://www.mlst.net) for the GASM/*emm44* species, isolated form noninvasive cutaneous infections. We identified a Tn916-like element inserted in the *srtB* gene, which disrupts the lantibiotic streptin locus (8). This mobile element (length 20.7 kb), assigned as Tn6253 (http://www.ucl.ac.uk/eastman/research/departments/microbial-diseases/tn), contains the *tetM* gene encoding for class M tetracycline resistance and a 2.6 kb Group II intron inserted within Tn916-*orf16* (9).

The genomic modifications described in the STAB901 strain provide insights into the functional characterization of the virulence of an epidemic clone and are under investigation.

### Nucleotide sequence accession number.

The complete genome and annotated sequence of *S. pyogenes* strain STAB901 has been deposited in the NCBI under the accession number CP007024.
